# Intestinal Obstruction among Patients Admitted in the Department of Surgery of a Tertiary Care Centre: A Descriptive Cross-sectional Study

**DOI:** 10.31729/jnma.7273

**Published:** 2022-04-30

**Authors:** Sajana Poudel, Sagar Panthi, Swotantra Gautam, Siddhartha Bhandari, Bharosha Bhattarai, Sandip Pokharel, Swastika Sedhai, Manoj Ghimire, Bhawani Khanal, Brikh Raj Joshi

**Affiliations:** 1 Department of Surgery, B.P. Koirala Institute of Health Sciences, Dharan, Sunsari, Nepal; 2 Department of Internal Medicine, Institute of Medicine, Maharajgunj, Kathmandu, Nepal; 3 Department of Surgery, Kathmandu University School of Medical Sciences, Dhulikhel, Kavre, Nepal; 4 Department of Surgery, Patan Academy of Health Sciences, Lagankhel, Lalitpur, Nepal; 5 Department of Surgery, Lumbini Provincial Hospital, Butwal, Rupandehi, Nepal

**Keywords:** *intestinal obstruction*, *large intestine*, *small intestine*, *surgery*

## Abstract

**Introduction:**

Although intestinal obstruction is a very common surgical emergency, there is a dearth of evidence regarding its prevalence at our institute. The objective of this study is to find out the prevalence of intestinal obstruction among patients admitted to the Department of Surgery of a tertiary care centre.

**Methods:**

A descriptive cross-sectional study on a total of 6735 admitted patients' in Department of Surgery a tertiary care centre was conducted from 1^st^ January, 2014 to 31^st^ March, 2015. Data were collected retrospectively with ethical approval from Institutional Review Committee (Reference number: 106/071/072). All patients admitted to the surgery ward of the hospital with an age of 18 and above were included in the study. Convenience sampling was used. The data were recorded and analyzed using Microsoft Excel and Statistical Package for Social Sciences version 16.0. Point estimate at 95% Confidence Interval was calculated along with frequency and proportion for binary data.

**Results:**

Out of the 6735 admitted cases, the prevalence of intestinal obstruction among the admitted patients in the surgery department of the tertiary care centre was found to be 100 (1.48%) (1.19-1.77 at 95% Confidence Interval). The most common presentations were pain in the abdomen 93 (93%), vomiting 74 (74%), and abdominal distension 55 (55%).

**Conclusions:**

The prevalence of intestinal obstruction in our study was lower than the similar studies done in similar settings.

## INTRODUCTION

Intestinal obstruction (IO) is one of the most common presentations to the emergency department, accounting for around 1.90% to 16% of all surgical admissions for acute abdomen cases.^1-3^ Abdominal pain, distension, vomiting, and constipation are some cardinal features of IO.^4^

A delay in the diagnosis and treatment in these cases invites devastating consequences like bowel ischemia secondary to vascular compromise, necrosis, perforation, sepsis, or even death.^5^ Although IO is a very common surgical emergency, there is a dearth of evidence regarding the actual disease burden, frequencies of the common underlying conditions, clinical presentation, and its complications and management at our institute.

The objective of this study is to find out the prevalence of IO among patients admitted to the Department of Surgery of a tertiary care centre.

## METHODS

A descriptive cross-sectional study was conducted in the Department of Surgery, B.P. Koirala Institute of Health Sciences, Dharan, Nepal from 1^st^ January, 2014 to 31^st^ March, 2015. Data were collected retrospectively with ethical approval from Institutional Review Committee (Reference number: 106/071/072). All patients admitted to the surgery ward of the hospital with an age of 18 and above were included in the study. Patients below 18 years of age, those with an incomplete set of information or misplaced files were excluded from the study. Convenience sampling method was used and the sample size was calculated as below:

n = (Z^2^ × p × q) / e^2^

  = (1.96^2^ × 0.0987 × 0.9013) / 0.02^2^

  = 2401

Where,

n = minimum required sample size
Z = 1.96 at 95% Confidence Interval (CI)
p = prevalence of intestinal obstruction, 9.87%^2^
q = 1-p
e = margin of error, 2%

Since convenience sampling was used in the study, doubling the sample size, we got 4802. However, 6735 cases were taken. Out of the total 6735 patients admitted to the surgery department, the files of 100 patients diagnosed with IO were retrieved in their complete form. Data regarding the demographic profile, clinical presentation, general physical examination, per abdominal examination, digital rectal examination (DRE), systemic examination findings, investigations Ultrasonography (USG), X-ray abdomen, Contrast-Enhanced Computed Tomography (CECT) abdomen] and type of management done (conservative or surgical and if surgical, type of operation done) were recorded in a predesigned proforma and analyzed using Microsoft Excel and Statistical Package for Social Sciences version 16.0. Point estimate at a 95% Confidence Interval and descriptive statistics were interpreted as frequencies, percentages.

## RESULTS

**Figure 1:**
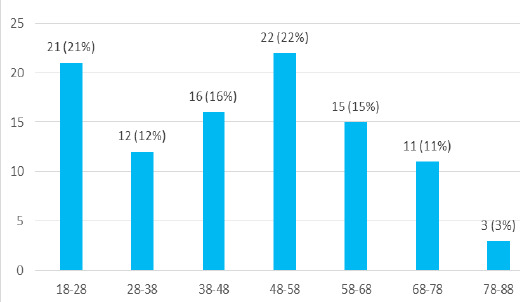
Age distribution of IO patients (n = 100).

Out of the 6735 admitted cases, the prevalence of IO among the admitted patients in the surgery department of the tertiary care centre was found to be 100 (1.48%) (1.19-1.77 at 95% Confidence Interval). Among the 100 patients taken into consideration, 65 (65%) were male and 35 (35%) were female with a male to female ratio of 1.85:1. Most of the paients were beweeen 48-58 years of age. Most of the patients were from Sunsari district 22 (22%) followed by Saptari 16 (16%) and Jhapa 16 (16%) (Figure 1).

**Figure 2:**
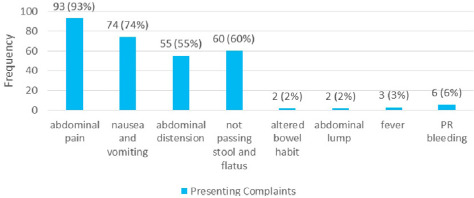
Presenting complaints among IO patients (n= 100).

History of previous abdominal surgery was present in 40 (40%) cases, of which the most commonly performed previous surgeries were exploratory laparotomy 35 (35%), gynaecological surgery 22 (22%) and appendectomy 17 (17%). Likewise, 5 (5%) of them had a past history of pulmonary tuberculosis and 3 (3%) had diabetes mellitus. The inquiries into their personal history revealed that 35 (35%) of the respondents smoked tobacco, 36 (36%) consumed alcohol, 22 (22%) were vegetarians and 78 (78%) had a mixed diet. The most common presenting complaint was abdominal pain 93 (93%) followed by nausea/ vomiting 74 (74%), not passing stool or flatus 60 (60%) and abdominal distension 55 (55%) (Figure 2).

The most common findings on physical examination were abdominal tenderness 91 (91%), abdominal distension 63 (63%) and an absence of bowel sound 7 (7%). Digital Rectal Examination (DRE) was performed on all the patients with 92 (92%) of them having no abnormal findings, 4 (4%) having a suspected intraluminal mass, 3 (3%) revealing ballooning of the rectal mucosa, and 1 (1%) patient revealing traces of blood in the examiner's finger after DRE. The commonest investigation performed on the cases was abdominal X-ray 100 (100%) followed by USG 89 (89%) and CECT abdomen 3 (3%).

**Figure 3:**
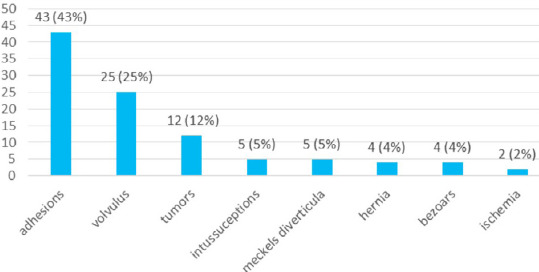
The common underlying conditions of IO (n= 100).

Looking at the presentation of IO, 65 (65%) cases had a subacute presentation and the remaining 35 (35%) had an acute presentation. The commonest underlying condition of IO was postoperative adhesion 43 (43%) followed by volvulus 25 (25%) and malignant obstruction 12 (12%) (Figure 3).

A nearly equal proportion of the patients received conservative management 49 (49%) and surgical management 51 (51%). The commonest performed operation was adhesiolysis 13 (25.49%), release of band 8 (15.68%), resection of sigmoid colon with double-barrel colostomy 6 (11.76%) and resection of ileum & anastomosis 6 (11.76%) (Table 1).

**Table 1:** Common surgical operations done among IO patients (n= 51).

Surgical operations performed	n (%)
Adhesiolysis	13 (25.49)
Release of band	8 (15.68)
Resection of sigmoid colon with double-barrel colostomy	6 (11.76)
Resection of ileum and anastomosis	6 (11.76)
Resection of colon and anastomosis	5 (9.80)
Derotation of terminal ileum	4 (7.84)
Resection of the ileal segment with double barrel ileostomy	3 (5.88)
Intraluminal breakage of bezoars	2 (3.92)
Derotation of sigmoid volvulus with double-barrel sigmoid colostomy	2 (3.92)
Ileal resection with double-barrel sigmoid colostomy	1 (1.96)
Sigmoid loop colostomy	1 (1.96)

Among the operated patients, small bowel obstruction was found in 29 (56.86%) and large bowel obstruction in 22 (43.13%) cases. The commonest intraoperative finding was adhesions and bands causing constriction and kinking in the small intestine 18 (35.29%) followed by sigmoid volvulus 8 (15.68%). Among the operated patients, 14 (27.45%) had postoperative complications, namely 5 (9.80%) respiratory tract infections, 3 (5.88%) wounds infections, 3 (5.88%) electrolyte disturbance and 3 (5.88%) having other complications.

## DISCUSSION

The prevalence of IO in our study was found to be 1.48% which is lesser than that of a study done in South India (1.90%) and in Ethiopia (4.80%) but very low compared with that of a study done in East India (9.87%).^1,2,6^ Our study showed a male predominance (65%) in patients presenting with IO with a male to female ratio of 1.85:1 which is also comparable to the studies done in African countries: Nigeria (Male:Female= 2:1) and Uganda (Male:Female= 2.6:1); but lesser as compared to two independent studies done in South India (Male: Female= 4:1) and East India (Male:Female= 3:1).^12,7,8^ The most common age group affected according to our study was 48-58 years, which is consistent with the studies done in East India (41-50 years) and South India (51-60 years).^1,2^

The triad of presentation of IO according to our study was pain abdomen (93%), vomiting (74%), and abdominal distension (65%) which are quite similar to the findings of a study done in South India in the same order of presentation.^1^ However, in a study done in East India, abdominal distension was found to be the commonest presentation of IO (93%) followed by vomiting (91%) and constipation (82%).^2^

In our study, 40% of the patients had a previous history of abdominal surgery, with exploratory laparotomy being the commonest one (35%), followed by gynaecological procedures (22%) and appendectomy (17%). An explanation could be due to the extreme manipulation of the bowel during exploratory laparotomy and the relatively common occurrence of gynaecological and appendectomy surgeries. Similar results were also seen in a study done in the United States of America where the incidence of previous surgery was 63%, however, in that study, colorectal surgery (34%) was the commonest one followed by gynaecological surgery (28%) and exploratory laparotomy (20%).^9^

Our study reported small bowel obstruction in 56% of the patients studied which is lower when compared to the findings of a study done in Athens (76%).^10^ Postoperative adhesions (43%) were the commonest underlying condition of obstruction found in our study which is comparable to other studies in South India (40%), Pakistan (41%) and the United States of America (54%).^1,11,12^ The second most common underlying condition in our study was volvulus (25%) which is contrasting with those done in South India and East India where the obstructed hernia was found to be the second most common underlying condition of IO.^1,2^ This difference could be explained owing to the awareness of the public and also to the fact that hernias are electively repaired because of which, the obstructed hernias are becoming less common. Malignant obstruction secondary to tumours (12%) was the third most common underlying condition in our study followed by other causes such as intussusception, band by Meckel's diverticula, hernia, bezoars etc. which were almost similar to the study done in Turkey where adhesions accounted for 48%, sigmoid volvulus 15.50% and tumours 20%.^13^

In our study, 49% of patients were managed conservatively while 51% needed surgical intervention. In a study done in Turkey, 58.70% were treated conservatively.^13^ Respiratory tract infections (9.80%) were the most common postoperative complication in our study followed by wound infections (5.88%) and electrolyte imbalance (5.88%) with a fairly substantial number of patients (72.55%) having no postoperative complications. This is similar to the findings of the study done in South India where septicemia was found in 10% of cases, respiratory tract infections in 4% and wound infections in 4%.^1^

Since, all the case files of IO could not be retrieved as they were incomplete or misplaced in the record section, the actual disease burden of IO could not be estimated in this study.

## CONCLUSIONS

The prevalence of IO among admitted patients in the Department of Surgery at our institute was lower than the similar studies done in similar settings. Pain abdomen, vomiting and abdominal distension comprised the triad of clinical presentation. The common underlying conditions were postoperative adhesions, sigmoid volvulus and tumours. An equal proportion of patients received conservative and operative management with a substantial proportion of operated patients not having any postoperative complications.
